# mRNA Vaccines in Melanoma Immunotherapy—A Narrative Review

**DOI:** 10.3390/cells15030298

**Published:** 2026-02-05

**Authors:** Paulina Plewa, Maciej Ćmil, Filip Lewandowski, Agata Poniewierska-Baran, Andrzej Pawlik

**Affiliations:** 1 Department of Physiology, Pomeranian Medical University, 70-111 Szczecin, Poland; paulina.plewa@pum.edu.pl (P.P.); agata.poniewierska-baran@usz.edu.pl (A.P.-B.); 2 Institute of Biology, University of Szczecin, 71-412 Szczecin, Poland; filip.lewandowski@usz.edu.pl

**Keywords:** melanoma, therapeutic mRNA vaccines, delivery systems

## Abstract

Melanoma is one of the most aggressive forms of cancer and the leading cause of death related to skin disease. Recent years have seen a significant increase in the number of cases of this type of cancer, underscoring the need to develop effective therapeutic strategies to control it. One of the most promising research directions in this field is anticancer immunotherapy, particularly the use of vaccines aimed at enhancing the body’s cellular immunity. Among the modern methods of this type, mRNA-based vaccines are prominent, gaining increasing importance as a potential tool in cancer therapy. Their main advantages include a relatively rapid and flexible production process, low production costs, and the ability to induce both humoral and cellular immune responses. Despite their numerous advantages, therapeutic mRNA vaccines also pose a number of scientific and technological challenges. These primarily concern the stability of mRNA molecules and their effective delivery to target cells. In this context, delivery systems such as lipid nanoparticles (LNPs) play a key role, protecting mRNA from degradation and facilitating its transport into the cell cytoplasm. Alternatively, systems based on biodegradable polymers are also being developed, which can provide controlled mRNA release and additional biocompatibility. However, before therapeutic mRNA vaccines become a routine component of cancer therapy, extensive clinical trials and a thorough understanding of their mechanisms of action are necessary. This paper provides an overview of the current knowledge regarding the structure and delivery methods of therapeutic mRNA vaccines, with a particular emphasis on their use in melanoma therapy. The results of clinical trials to date are also presented and the challenges associated with implementing this form of therapy in medical practice are discussed.

## 1. Introduction

Melanoma is considered the most aggressive type of skin cancer [1]. It results from the malignant transformation of melanocytes, pigment-producing cells. Melanoma can occur in various anatomical locations, including the skin, mucous membranes, the choroid, and the meninges [2]. The statistics are alarming; melanoma causes nearly 75% of deaths from skin cancer. The incidence of malignant melanoma is increasing, causing a serious socio-economic problem. The last GLOBOCAN report from 2022 indicated that melanoma was the 17th most common cancer—globally, 331,722 people were diagnosed with melanoma and 58,667 died from this disease [3,4]. Melanoma is one of the more common cancers among young and middle-aged individuals, which distinguishes it from other solid tumors detected predominantly in older adults. Traditional cancer therapies, such as chemotherapy, radiotherapy, and surgery, have limited efficacy and are associated with numerous side effects [5].

Although advances in melanoma diagnostics have enabled earlier detection of the disease, the prognosis for patients with advanced or metastatic melanoma remains poor. One of the most dangerous features of malignant melanoma is its ability to rapidly metastasize. To estimate the risk of metastasis, factors such as the depth of invasion of the primary tumor, the presence of ulceration, micrometastases in regional lymph nodes, and the number of mitoses in thin tumors are considered [6]. The stage of cancer advancement determines the choice of the optimal therapeutic strategy [7]. Current treatment options include surgery, chemotherapy, radiotherapy, and immunotherapy. Cancer vaccines are immunotherapeutics and currently administered to clinical trial participants. Therapeutic cancer vaccines offer a novel strategy that activates the immune system to target specific cancer cells. It remains a significant challenge, as cancer cells can evade detection by the immune system [8,9,10,11]. Currently, several therapeutic mRNA vaccines in clinical trials are being tested in clinical trials in melanoma but also other cancers, including lung cancer [12], pancreatic and colorectal cancer [13], and head and neck cancer [14].

This article summarizes available data on the structure, mechanisms of action, and methods of administration of mRNA vaccines in preclinical models and clinical trials, with a particular focus on their use in melanoma therapy. We analyzed their therapeutic potential, immunological efficacy, and challenges related to safety and implementation in clinical practice. A literature review was conducted by searching the PubMed and Scopus databases, covering publications from 1998 to 2024. The search was performed using keywords and their combinations in English. The main search terms included: melanoma, mRNA vaccines, mRNA-based therapy, cancer immunotherapy, and delivery systems. Synonyms and related terms were also included to maximize the search scope. Only full-text original and review articles published in peer-reviewed scientific journals in English were included in the analysis. Conference proceedings, master’s and doctoral theses, and publications in languages other than English were excluded.

## 2. Melanoma-Associated Antigens

**Figure 1:**
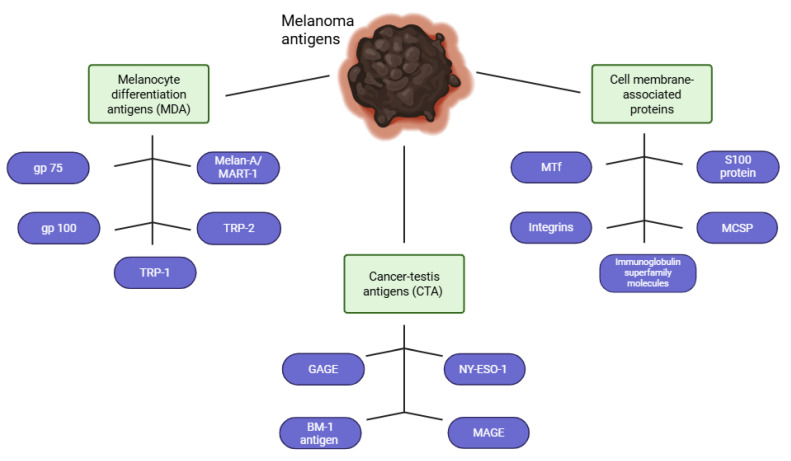
The diagram illustrates the major groups of melanocyte differentiation antigens (MDAs), cancer-testis antigens (CTAs) and cell membrane-associated proteins. MDAs include tyrosinase, tyrosinase-related proteins TRP-1 and TRP-2, Melan-A/MART-1 antigen, glycoprotein 75 (gp75), and glycoprotein 100 (gp100). Cell membrane-associated proteins include integrins, immunoglobulin superfamily molecules, melanotransferrin (MTf), S100 protein, and chondroitin sulfate proteoglycan (MCSP). The CTA group includes MAGE, GAGE, BABe, and NY-ESO-.Created in BioRender. Plewa, P. (2025). https://BioRender.com/yefsodp (accessed on 19 November 2025).

As melanoma progresses, cancer cells exhibit the ability to express various molecules, both on the surface and intracellularly. These molecules include tumor-specific antigens and physiological proteins that naturally occur in healthy tissues but are ectopically expressed. Tumor-specific antigens can be used as immunotherapy targets. Based on their biological properties, melanoma antigens can be divided into several groups: melanocyte differentiation-associated antigens, cancer-testis antigens and cell membrane proteins (Figure 1).

Melanocyte differentiation antigens (MDAs) are normal and non-mutated proteins expressed in melanoma cells, as well as in normally developing melanocytes during various stages of differentiation. These antigens are located in the areas of the cell responsible for melanin synthesis, including melanosomes and specialized organelles, making them highly specific markers of melanocyte differentiation [15]. Furthermore, the presence of tumor-infiltrating lymphocytes [16], which can recognize antigens associated with melanocyte differentiation, makes these molecules promising targets for immunotherapy [17]. An MDA includes tyrosinase, tyrosine-related proteins (TRP-1 and TRP-2), melanoma antigen recognized by T cells (Melan-A/MART-1) [18], glycoprotein 75 (gp75) [19], and glycoprotein 100 (gp100) [15] (Figure 1).

Cancer-testis antigens (CTAs) are associated with approximately 80 proteins, each belonging to a specific family. Under physiological conditions, these antigens are expressed in trophoblasts, germ cells of the testis during various stages of spermatogenesis, and medullary epithelial cells of the thymus [20,21]. Furthermore, some antigens are expressed in somatic tissues only during the developmental period [20]. In adult humans, the activity of these antigens is usually limited to germ cells, and their re-expression may indicate the process of carcinogenesis. This phenomenon has been observed in various types of cancer, including melanoma [15]. CTAs include melanoma-associated antigen (MAGE), G antigen (GAGE), BM-1 antigen, and NY-ESO-1 [21,22] (Figure 1).

Cell membrane-associated proteins are attractive immunotherapy targets because of their increased expression in melanoma cells [23]. Among the best-characterized and most frequently analyzed receptors are integrins, immunoglobulin superfamily molecules, melanotransfetin (MTf), S100 protein, and melanoma chondroitin sulfate proteoglycan (MCSP) [15] (Figure 1).

Although mRNA vaccine and immunotherapy technologies offer a powerful way to stimulate targeted immune responses, their effectiveness can be limited by challenges such as mRNA instability, variable delivery efficiency, and the possibility of insufficient or short-lived antigen expression. Additionally, individual differences in immune responsiveness may reduce the consistency of therapeutic outcomes in cancer patients.

## 3. Therapeutic mRNA Vaccines

Therapeutic mRNA vaccine and immunotherapy technology involves the use of mRNA sequences encoding specific antigens which, when expressed in host cells, induce a targeted immune response, enabling the prevention and treatment of diseases, including cancer. The mRNA molecules contained in vaccines work in the same way as the natural mRNA found in human cells, providing a template for protein production. The mechanism of therapeutic mRNA vaccines’ action is simple, yet revolutionary, based on the transfer of genetic information regarding specific antigens, such as proteins (viral, bacterial or cancer), into host cells that read the genetic information and produce the protein according to the coding regions contained in the mRNA. When cells begin to produce the antigenic protein, the body responds by activating the immune system. The immune system recognizes these proteins as foreign and produces antigen-specific antibodies, while mobilizing T cells, which are intended to attack and destroy both the pathogen proteins and the infected host cells [24].

Two types of therapeutic mRNA vaccine constructs are available: nonreplicating mRNA (NRM) and self-amplifying mRNA (SAM) [24]. The structure of mRNA vaccines consists of a single-stranded oligonucleotide featuring a 5′ cap, a 3′ poly(A) tail, and an open reading frame (ORF) flanked by untranslated regions (5′ and 3′ UTRs). Currently, a preventive or therapeutic mRNA vaccine is typically administered by injection. Structurally, mRNA vaccines are optimized to ensure stability and efficient translation [25,26]. One major challenge for therapeutic mRNA vaccines is the rapid degradation of naked mRNA, which is inherently unstable and is highly susceptible to degradation by extracellular ribonucleases (RNases). The effectiveness of the vaccine depends on the effective delivery of mRNA into the cell, its translation by ribosomes and the synthesis of the encoded protein. Enhancements through mRNA engineering have substantially increased and extended the functional half-life mRNA. The therapeutic mRNA vaccines are delivered into cells by transfecting agents or formulated with delivery vehicles. Efficient mRNA delivery is a critical factor for the therapeutic success of mRNA vaccines, and an efficient delivery system helps mRNA vaccines achieve their full therapeutic potential [25,26]. Importantly, in terms of vaccine therapeutic use, the composition may include two or more mRNAs coding for different proteins antigens or even long peptides [27].

### 3.1. Delivery Systems in mRNA Therapy

**Figure 2:**
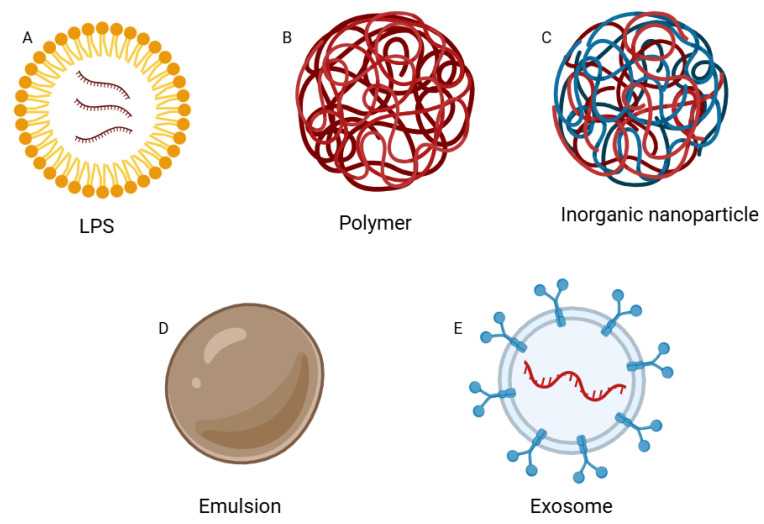
Figure illustrating various nanoparticle-based delivery systems. (**A**)—Liposomes are spherical vesicles composed of a lipid bilayer, commonly used as carriers for drugs and genetic material due to their high biocompatibility and ability to protect the payload from degradation. (**B**)—Polymeric nanoparticles have the ability to regulate their physicochemical properties, allowing for controlled and sustained release of therapeutic cargo. (**C**)—Inorganic nanoparticles possess unique optical and magnetic properties that find applications in both diagnostics and targeted therapies. (**D**)—Emulsions are systems of two immiscible liquids, primarily used to transport hydrophobic compounds. (**E**)—Exosomes, natural vesicles secreted by cells, play a crucial role in intercellular communication. Due to their biological origin, they can be modified to carry mRNA, making them promising carriers for immunotherapy. Their similarity to pathogens makes them effective in inducing the body’s immune response. Created in BioRender. Plewa, P. (2025). https://BioRender.com/2txg7nh (accessed on 19 November 2025).

Various strategies have been developed to facilitate the passage of therapeutic mRNA vaccines through the cell membrane and their delivery to target cells. Difficulties with membrane passage stem from the size, instability, and polarity of mRNA. mRNA-based preparations are most often absorbed via endocytosis, as they are unable to penetrate the cell membrane by passive diffusion on their own. Delivery systems can significantly reduce degradation of mRNA and protect against hydrolysis. Furthermore, they can enhance the mRNA’s entry into cells and escape from the endosome, thus improving transport to lymphatic organs and cellular targets. Nanodelivery systems can be functionalized by attaching ligands, antibodies, or peptides that bind to specific receptors on the surface of target cells. Furthermore, some nanoparticles can act as adjuvants, substances that enhance the body’s immune response. Nanoparticles provide a universal platform that can be easily adapted to different mRNA sequences and different types of vaccines. Changing only the antigen-coding fragment allows for the rapid development of a vaccine against a new pathogen without the need to modify the entire delivery system. Delivery systems facilitate the acquisition of the correct antigen and the induction of an immune response [28]. There are several delivery systems, based on lipids, polymers, exosomes, emulsion and inorganic nanoparticles (Figure 2).

#### 3.1.1. Lipid-Based Delivery System

Lipid-based mRNA are currently among the best-characterized and most clinically advanced mRNA delivery platforms, making them a dominant strategy in translational and clinical research. Their effectiveness stems from their high capacity for mRNA encapsulation, protection from degradation, and efficient endosomal escape.

Liposomes are small vesicles whose membrane is similar to the of the cell membrane. They contain a closed phospholipid bilayer. Liposomes are the most classic type of lipid-bound carrier. They are characterized by relatively low toxicity, significant biocompatibility and ease of development, but their application may be limited by moderate stability and lower transfection efficiency compared to modern lipid nanoparticles [29]. Many of them have been extensively studied for their potential use in cancer treatment [28]. Lipoplexes, which are complexes of nucleic acid with liposomes, can also be used. This connection is possible due to the positive surface charge of cationic lipids, which electrostatically interacts with the negatively charged phosphate groups of RNA, leading to the formation of a stable complex. The most well-known are DOTMA (1,2-di-O-octadecenyl-3-trimethylammoniopropane) and DOTAP (1,2-dioleoyl-3-trimethylammoniopropane) [30,31]. Their activity and appropriate use are related to obtaining the correct overall charge of particles, appropriate lipid content and a consistent ratio of cationic lipids to mRNA [32]. Although DOTAP is a relatively good mRNA carrier, it shows toxicity in vivo [31]. Ionizable LNPs are the most common in vivo mRNA delivery system. They primarily consist of four components: an ionizable or cationic lipid, phospholipids, cholesterol, and lipid-linked PEG. Cholesterol acts as a stabilizing agent, while phospholipids act as a supporting agent, enabling the formation of a lipid bilayer structure. PEG prolongs the circulation period of LNPs by preventing mRNA binding to plasma proteins. Their advantage over other lipid systems is their neutral charge under physiological conditions and the ability to protonate in the endosomal environment [33]. In the case of virofenib-resistant melanoma, nanoliposomes containing glycosylated PEG-linked BRD4 PROTACs may be an effective treatment option [34]. Liu et al. [35] developed a strategy for delivering cytokine-encoding mRNA to the TME using novel LNPs. Studies have shown that diaminolipid-based nanoparticles effectively deliver mRNA, enabling the expression of cytokines both in vitro and in vivo. The most significant inhibition of melanoma growth was observed with the combined delivery of IL-12 and IL-27 mRNA.

#### 3.1.2. Polymer-Based Delivery System

Polymeric-based delivery system also known as polyplexes, represent a more diverse but less clinically advanced. These nanoparticles can be generated from both natural and synthetic polymers. Therefore, they can possess distinct properties that can be exploited for specific purposes [36]. Polymer-based delivery systems are based on electrostatic interactions between cationic polymers and negatively charged nucleic acids. This interaction creates a protective shield for mRNA molecules, preventing nucleic acid degradation by nucleases and increasing stability and transport efficiency. Compared to lipid-based delivery systems, polymer-bound carriers are characterized by reduced transfection efficiency and potential toxicity. Furthermore, they demonstrate significant potential for forming various nanostructures under aqueous conditions, as well as for long-term storage and lyophilization [37]. There are several polymers used in therapies: polyesters, dendritic polymers, PEI, and poly(amino acids).

Polyesters are characterized by appropriate properties in terms of biodegradability, biocompatibility and biological safety. The most commonly used are PLGA, PBAE, APE and PACE [38,39]. PLGA, or polylactic glycolic acid, exhibits adequate mechanical properties in processing. Moreover, they create a protective barrier around the mRNA molecule, protecting against enzymatic degradation and increasing the efficiency of delivery to cells. Furthermore, it can effectively cross the skin barrier, even into deeper melanoma lesions, while still enabling gradual release of the preparation [40]. Among the compounds tested is AD 3281, which has a strong ability to inhibit the MetAp2 enzyme. This enables the inhibition of cancer and endothelial cell proliferation as well as impairment of vessel formation in vitro. The combination of AD 3281 with PLGA improved its ability to penetrate cells, its uptake and enhanced its anticancer activity [41].

Dendrimers are a group of carriers characterized by significantly branched molecules with broad internal cavity structures and compact surface-active functional groups [29]. This structure enables much more efficient encapsulation of mRNA, thus providing protection against enzymatic degradation. Furthermore, dendrimers are characterized by lower toxicity and exhibit a relatively high rate of uptake and accumulation within solid tumors compared to free drugs [42]. Moreover, modifying their surface allows for a significant increase in cellular uptake [43]. A study conducted on cationic polyamidoamine dendrimer (amino-terminal PAMAM) as a carrier for the delivery of doxorubicin (DOX) and the immunoadjuvant cytosine–phosphate–guanine oligodeoxynucleotide (CpG ODN), used in the treatment of metastatic melanoma, demonstrated several disadvantages. PAMAM showed relatively low biocompatibility, significant toxicity and rapid clearance from the bloodstream due to the positively charged surface [44].

PEI, a cationic polymer, is associated with the delivery of nucleic acids based on electrostatic interactions. Its clinical application is limited due to low biocompatibility and biodegradability. In the case of poly(amino acids), which include poly(lysine), their role is related to the formation of a core–shell structure, thanks to the presence of amphiphilic block copolymers in their structure [45].

#### 3.1.3. Inorganic Nanoparticle-Based Delivery Systems

Inorganic nanoparticles encompass various types of particles, both metallic and non-metallic, which may represent a fairly broad platform for diverse drug delivery strategies [46]. Gold nanoparticles deserve attention, as they are characterized by the ability to selectively transport therapeutic substances to melanoma cells. This may positively impact the effectiveness of treatment [47]. The use of silver nanoparticles is also a suitable therapeutic strategy due to their properties, enabling more efficient transport of drugs and genetic material to specific regions in the human body [48].

#### 3.1.4. Extracellular Vesicles

Extracellular vesicles (EVs) are a diverse collection of membrane vesicles that delivery bioactive cargo and modulate cellular activity. They are naturally released from cells. EVs are surrounded by a lipid bilayer. EVs contain various cellular components, including proteins, lipids, and nucleic acids derived from the stem cell [49]. EVs associated with cancer have immunomodulatory properties, which can influence the functioning of immune cells. In particular, EVs secreted by melanoma cells can effectively inhibit the proliferation and survival of CD8^+^ T cells through a variety of mechanisms [50]. Moreover, due to their ability to cross biological barriers, their natural biocompatibility, and their properties that enable targeted delivery, EVs represent a promising platform for genetic material delivery systems in melanoma therapy [51].

### 3.2. Basics of mRNA Vaccine Technology and Their Advantages

Therapeutic mRNA vaccines offer several significant advantages over traditional vaccine platforms. The key advantages of mRNA vaccines include the lack of integration into the host genome, the possibility of rapid and mass production at relatively low costs, and the ability to induce a long-lasting immune response. There are several key steps in the production and characterization of mRNA vaccines, with the process beginning with the identification of tumor antigens with high specificity for a given tumor, including so-called neoantigens, which arise as a result of somatic mutations and are potentially recognized by CD8^+^ T cells. Companies such as BioNTech and Moderna are examining mRNA vaccines targeting specific mutations or neoantigens characteristic of melanoma. These antigens are potential targets for specific anti-melanoma immunotherapy. In some cases, mRNA vaccines encode multiple antigens simultaneously, which aims to increase the efficacy of the immunotherapy. Preclinical and early-phase clinical trials have shown that these vaccines induce a cytotoxic T-cell response against melanoma cells and, when combined with checkpoint inhibitors, may increase the efficacy of the therapy. It is worth noting that BioNTech and Moderna are not the only companies working on melanoma vaccines; research is also underway at other academic centers and biotechnology companies worldwide on mRNA, peptides, and viral vectors encoding melanoma antigens. However, BioNTech and Moderna are pioneers in mRNA platforms, which enable the rapid design of individual and multi-antigen vaccines against melanoma [52,53].

The next step involves designing and synthesizing the mRNA. The coding sequence for the selected antigen is designed and optimized for translation efficiency in human cells, molecular stability, and minimization of innate immune response by avoiding motifs recognized by Toll-like receptors (TLR3, TLR7/8) and other RNA receptors. To achieve the desired characteristics, a combination of various strategies is used: codon optimization, introduction of modified nucleotides (e.g., pseudouridine or N1-methylpseudouridine), modification of the 5′ cap (cap1), and 3′ polyadenylation, which increases translation efficiency and mRNA stability in target cells. The mRNA is synthesized in vitro using RNA polymerase enzymes and subjected to rigorous purification filtration to remove double-stranded fragments that could induce undesirable immunogenicity [53,54].

Then in mRNA vaccine design involves formulating and creating a lipid envelope. The mRNA is often encapsulated in LNPs, which protect it from enzymatic degradation and enable efficient delivery to cells. Industrial production of mRNA vaccines requires rigorous quality control and sterility, including assessment of mRNA integrity, lipid purity, encapsulation efficiency, and final product stability. The process involves validation of mixing, encapsulation, filtration, and lyophilization steps, as well as precisely defined storage conditions at ultralow temperatures (e.g., −70 °C), necessary due to the sensitivity of mRNA to thermal degradation. Furthermore, real-time monitoring of quality parameters is increasingly being implemented, enhancing the safety and reproducibility of clinical-scale production.

The rate and scale of protein expression with mRNA are typically higher than with DNA vaccines due to bypassing the need for nuclear transport, transcription, and transcription factor dependence, allowing direct and multiple translations in the cytoplasm. Furthermore, mRNA does not modify the host genome, as mRNA does not integrate into chromosomal DNA but only temporarily introduces genetic information into cells [54].

Recent successes of mRNA vaccines in the fight against COVID-19 have sparked interest in their potential in cancer immunotherapy. Interestingly, in a single case report, a cancer patient who received the second and subsequent doses of the Pfizer–BioNTech mRNA vaccine against SARS-CoV-2 (the mRNA strands encapsulated in LNPs) experienced regression of numerous metastatic skin melanoma nodules [55]. Only 2 days after vaccination, all melanoma skin nodules became painful and significantly reduced in size. These data suggest that the therapeutic mRNA vaccine may exert antitumor and apoptotic effects by stimulating Toll-like receptors TLR3, TLR7 and TLR8 and (subsequently) the nuclear factor kappa-light-chain-enhancer pathway of activated B-cell nonlymphocytic immune effector cells [56,57].

mRNA vaccine technology is therefore an innovative therapeutic approach aimed at treating melanoma and other cancers, involving the use of advanced biotechnological tools to precisely stimulate the immune system against existing cancer [58,59]. Future developments include the development of strategies vaccines, combining mRNA therapy with other immunotherapy methods, and further research into personalized treatment.

### 3.3. The Mechanisms of Action of mRNA Vaccines in the Context of Melanoma

mRNA-based vaccines represent one of the most promising modalities in tumor-specific immunotherapy, particularly for malignancies with high mutational burden such as cutaneous melanoma, which frequently exhibits over 10 mutations per megabase and generates a diverse array of immunogenic neoantigens [60,61]. By encoding either patient-specific neoantigens or shared melanoma-associated antigens (tyrosinase, TPTE), these vaccines—unlike classical peptide-based approaches—ensure authentic antigen production in situ with efficient presentation through both MHC class I and class II pathways, enabling rapid personalization and induction of robust cytotoxic and helper T-cell responses [28,62,63,64].

The process begins with whole-exome and transcriptome sequencing of the patient’s tumor tissue and matched normal tissue. Bioinformatic analysis identifies nonsynonymous mutations capable of generating neoepitopes with high predicted affinity for the patient’s MHC (HLA) alleles. Selected neoantigen-encoding sequences are assembled into poly-epitope constructs and transcribed into stabilized mRNA (frequently incorporating modified nucleosides such as N^1^-methyl-pseudouridine to enhance translational efficiency and reduce innate immunogenicity) [24,28,60,61]. The resulting mRNA molecules are encapsulated in lipid nanoparticles (LNPs), which protect them from nucleases and facilitate efficient uptake by antigen-presenting cells [7,65].

Contemporary mRNA vaccines employ nucleoside-modified mRNA, purified by high-performance liquid chromatography, with a 5′ cap, optimized untranslated regions, and a poly(A) tail [24,66]. The mRNA is encapsulated within ionizable LNPs composed of ionizable cationic lipids, PEGylated lipids, cholesterol, and helper phospholipids (e.g., DSPC or phosphatidylserine in certain formulations). These LNPs, typically 80–150 nm in diameter with near-neutral surface charge, facilitate efficient lymphatic drainage following intramuscular or intradermal injection [7,67]. Professional antigen-presenting cells, particularly conventional type 1 dendritic cells (cDC1s) at the injection site or in draining lymph nodes, internalize the LNPs via macropinocytosis, phagocytosis, or receptor-mediated endocytosis involving mannose receptor or DC-SIGN. Upon endosomal acidification, protonation of ionizable lipids disrupts the endosomal membrane, releasing mRNA into the cytosol with high efficiency [7,67,68]. In the cytosol, the mRNA is translated by host ribosomes into full-length proteins that undergo native post-translational modifications, including glycosylation and proper folding [68]. This cytosolic expression directs antigens primarily into the MHC class I pathway: proteasomal degradation produces peptides that are transported via transporter associated with antigen processing (TAP) into the endoplasmic reticulum, loaded onto MHC class I molecules, and presented to CD8^+^ T cells. Concurrently, antigen access to the MHC class II pathway occurs through autophagy or vesicular routing, enabling CD4^+^ T-helper cell activation [69,70]. Processing of the translated antigens, combined with maturation signals (detailed below), induces profound phenotypic changes in the transfected dendritic cells: upregulation of the chemokine receptor CCR7 and other migration-associated molecules, which direct the mature, antigen-loaded dendritic cells to migrate from the injection site or peripheral tissues to the draining regional lymph nodes. This CCR7-dependent migration is essential for efficient encounter with naive T cells in secondary lymphoid organs [28,63,71].

The mRNA itself serves as an intrinsic adjuvant. Despite nucleoside modifications that attenuate excessive inflammation, residual recognition occurs via endosomal Toll-like receptors 7/8 (TLR7/8) and cytosolic sensors RIG-I/MDA5, activating MyD88- and MAVS-dependent pathways that lead to NF-κB and IRF3/7 nuclear translocation [71]. This results in type I interferon (IFN-α/β) production, upregulation of costimulatory molecules (CD80, CD86, CD40), increased MHC expression, and secretion of proinflammatory cytokines such as IL-12 and TNF-α, establishing a Th1-biased microenvironment and providing the critical maturation stimulus for dendritic cells. Type I IFNs further induce chemokines CXCL9, CXCL10, and CXCL11, which recruit CXCR3-expressing effector T cells and enhance cross-presentation by other APCs [72,73]. In the T-cell zones of draining lymph nodes, mature dendritic cells present processed peptides on MHC class I and II molecules to naïve CD8^+^ and CD4^+^ T lymphocytes, leading to their clonal expansion and differentiation into polyfunctional effector cells secreting IFN-γ, TNF-α, granzyme B, and perforin [55,74]. CD4^+^ T-helper cells provide critical help via CD40L–CD40 interactions that further license dendritic cells and support the generation of central and effector memory T-cell populations [55]. Vaccine-induced T cells express tissue-homing receptors (e.g., CXCR3, CXCR5), facilitating their egress from lymph nodes and infiltration into melanoma lesions [75].

Upon tumor recognition, cytotoxic T lymphocytes release perforin to form membrane pores and granzymes to activate caspase cascades, inducing apoptosis in melanoma cells [74]. Tumor cell lysis liberates additional tumor antigens and damage-associated molecular patterns, promoting antigen spreading and recruitment of new T-cell clonotypes against epitopes not encoded in the original vaccine [75]. In the immunosuppressive melanoma microenvironment, vaccine-induced type I IFNs and T-cell-derived signals reprogram myeloid and stromal compartments. CXCL9/10/11 gradients overcome physical and chemokine barriers to T-cell infiltration, while IFN-γ suppresses immunosuppressive factors such as indoleamine 2,3-dioxygenase and TGF-β [71]. CD40 ligation drives IL-12 production, polarizing macrophages toward an M1 phenotype and activating natural killer cells. These changes shift immunologically “cold” tumors toward inflamed “hot” lesions with improved effector-to-regulatory T-cell ratios [73].

Specialized formulations augment these mechanisms. Phosphatidylserine-containing LNPs enhance antigen presentation to immune effectors, while protamine-complexed mRNA (as in RNActive platforms) stabilizes the construct and increases TLR7 engagement [76]. Co-delivery of cytokine-encoding mRNAs (e.g., IL-12, IFN-α) or immunomodulatory siRNAs directly reprograms the tumor microenvironment, and targeting ligands improve dendritic cell uptake and cross-presentation efficiency [77,78]. In ex vivo approaches, mRNA-electroporated autologous dendritic cells undergo controlled maturation and migrate to lymph nodes with high fidelity, bypassing delivery barriers entirely [79].

In summary, mRNA vaccines orchestrate a self-reinforcing cycle of LNP-mediated delivery, cytosolic antigen translation, dendritic cell maturation and CCR7-dependent migration to draining lymph nodes, innate adjuvanticity via type I IFN pathways, dual-arm T-cell priming in secondary lymphoid organs, tumor infiltration, and microenvironment remodeling that is ideally suited to the neoantigen-rich biology of melanoma. This multifaceted mechanism enables the immune system to recognize and eliminate melanoma cells with high specificity and durability.

## 4. Clinical and Translational Evidence for the Efficacy of Therapeutic mRNA Vaccines in Melanoma

The concept of mRNA-based cancer vaccines dates back to the late 1990s, when studies were initiated on ex vivo loading of DCs with mRNA encoding melanoma antigens, followed by reinfusion of such cells into patients [80]. In 2004, the first direct administration of an mRNA vaccine to patients was undertaken, which was protamine-protected mRNA encoding a set of six melanoma antigens (Melan-A/MART-1, MAGE-A1, MAGE-A3, tyrosinase, gp100, and survivin) together with the adjuvant GM-CSF [28,81]. Early phase I clinical trials demonstrated that this immunotherapeutic cannot be immunogenic, but the induction of antigen-specific T cells observed in a subset of patients. Although therapeutic response rates were low, isolated cases of complete tumor regression following mRNA vaccination with melanoma antigens were reported [28]. A key challenge, however, was the limited durability of the immune response and the high intrinsic immunogenicity of unmodified mRNA (triggering innate rather than the desired antigen-specific responses). This problem was addressed through chemical modifications of mRNA (e.g., replacement of uridine with pseudouridine), which markedly increased transcript stability and reduced unwanted innate immunogenicity [80]. These technological advances, initiated by the pioneering work of Katalin Karikó and colleagues in 2005, paved the way for the development of modern mRNA vaccine platforms [82].

Studies demonstrated the safety and strong immunogenicity of personalized neoantigen vaccines in patients with melanoma, showing multi-epitope CD4^+^/CD8^+^ responses, persistence of memory, and in some patients, absence of relapse. Among patients who did relapse, clinical responses to subsequent anti-PD-1 immunotherapy were associated with an expanded repertoire of neoantigen-specific TCRs [83].

BNT111 (BioNTech), the so-called FixVac vaccine, containing mRNA encoding four shared melanoma antigens (including NY-ESO-1, tyrosinase, MAGE-A3 and others), was delivered in lipid complexes (RNA-LPX) [28]. The MERIT trial, involving intravenous administration of the vaccine, demonstrated a favorable safety profile (mostly transient flu-like symptoms, with no dose limiting toxicities) [84]. Strong immunogenicity was observed with ~75% of patients developing CD4^+^ and/or CD8^+^ T cells that recognized at least one of the encoded antigens. The first signs of antitumor efficacy were also recorded; in the BNT111 monotherapy group, partial regressions (PRs) were achieved in 12% of patients. In contrast, in the cohort receiving the combination with a PD-1 inhibitor, the PR rate increased to 35% (6 of 17 patients, with disease stabilization in several additional cases). These results suggest a synergistic effect with immunotherapy. A randomized phase II trial of BNT111 in patients with advanced melanoma resistant to anti-PD-1 therapy is ongoing, comparing the vaccine in combination with cemiplimab against monotherapy [71].

The strongest clinical signal to date has come from the randomized phase IIb KEYNOTE-942 trial (NEJM 2024), in which the individualized neoantigen mRNA vaccine mRNA-4157 [85], combined with pembrolizumab, significantly reduced the risk of recurrence or death by ~44% (HR ~0.56; *p* = 0.0266) compared with pembrolizumab alone, with the benefit maintained at ≥30 months of follow-up. Patients’ clinical status was monitored systematically, according to a predefined schedule, including baseline assessment and regular follow-up visits. Assessment included physical examination, laboratory tests, and imaging studies such as computed tomography (CT) or magnetic resonance imaging (MRI) at study entry, then every 12 weeks for the first year after initiation of pembrolizumab treatment, and then every 12 weeks from months 12 to 24 and every 26 weeks from months 24 to 36. If relapse was suspected, histological confirmation was sought if tissue samples were available. For patients without clinical events, data on follow-up were censored based on the most recent documented disease assessment. At 18 months, the proportion of patients without relapse was 78.6% in the vaccine plus pembrolizumab arm versus 62.2% in the pembrolizumab alone arm. The benefit was observed across all subgroups, including both BRAF-mutant and BRAF-wild-type patients. Based on these results, phase III trials have already been initiated, and the vaccine is being tested globally in resected melanoma and in early-stage lung cancer. These results support the concept that mRNA vaccination complements PD-1 blockade by providing new targets for T-cells and reinforcing immunological memory [85].

mRNA-4157 (V940) is a personalized mRNA-based therapeutic strategy designed to induce an antitumor response by encoding a set of up to 34 neoantigens unique to a patient’s tumor. The immunological properties and mechanism of action of this therapy were evaluated in a phase I human study—KEYNOTE-603 (NCT03313778)—conducted in patients with completely resected cutaneous melanoma. In one cohort (designated Part D), 12 participants received combination therapy consisting of the mRNA-4157 vaccine and pembrolizumab. Immunological analyses demonstrated that the therapy led to the sustained induction of specific T cell responses recognizing the vaccine neoantigens and an increase in the number of cytotoxic CD8^+^ and CD4^+^ effector T cells (Table 1) [86].

Mechanistically, mRNA vaccines (I) increase the ‘antigenic load’ and diversity of epitopes recognized by TCRs (including novel high-avidity clones) [87], (II) activate DCs (particularly cDC1) through innate signals (type I IFN) [30,52], (III) promote the NK–cDC1 cascade [88] and the production of CXCL9/10 within the tumor, thereby facilitating the recruitment and retention of effector CD8^+^/CD4^+^ cells [87,88] and (IV) induce and sustain T-cell memory that underpins control of minimal residual disease [89,90]. Translational studies in vaccinated patients have demonstrated tumor infiltration by TCR clones specific for vaccine epitopes, as well as the broadening of responses to non-vaccine epitopes, indicating the in situ amplification of the cancer–immunity cycle [91,92].

Other platforms are also evaluating alternative strategies in parallel, with early studies underway [91]. One example is CV8102 (CureVac), a non-coding RNA administered intratumorally (or intramuscularly), serving as an endogenous adjuvant by activating TLR7/8 and RIG-I in immune cells within the TME. In a phase I study in PD-1-refractory melanoma, CV8102 demonstrated a favorable safety profile and preliminary efficacy signals: in a cohort of 30 patients treated with CV8102 plus anti-PD-1, ~17% achieved partial responses (5 patients), despite advanced, previously treated disease [93]. Another approach involves mRNA vaccines co-delivering additional immunostimulatory factors, for example, experimental formulations encoding both classical melanoma antigens and cytokines or co-stimulatory ligands. Clinical trials are ongoing with mRNA constructs delivering interleukins (IL-23, IL-36γ) and OX40L, aiming to reprogrammed the TME towards greater immunoreactivity. Such ‘combination’ mRNA vaccines, often administered together with a checkpoint inhibitors antibody, hold the potential to overcome resistance in tumors previously unresponsive to standard immunotherapy [94]. The mRNA platform is also being investigated in other types of cancer. CureVac has conducted early trials of mRNA vaccines in lung and prostate cancer, while BioNTech is developing FixVac programmers for prostate cancer (BNT112), HPV-positive head and neck cancer (BNT113) and others, employing similar RNA-LPX technology as in melanoma [71].

Combining mRNA vaccines with immune checkpoint inhibitors is currently a key strategy to enhance their clinical efficacy. mRNA vaccines prime and expand the pool of tumor-specific T cells, while blockade of PD-1 or CTLA-4 abolishes inhibitory signals in the TME, allowing for the full development of an antitumor response. Therefore, almost all current clinical protocols for mRNA vaccines in melanoma involve their use in combination therapy, and further studies are aimed at optimizing the treatment sequence and identifying patients who will benefit most from them [28,90,94,95,96].

## 5. Challenges and the Future of mRNA Therapy in Melanoma

The complexity of developing therapies is further compounded by the biological heterogeneity of melanoma, whose tumors differ significantly in their genetic and antigenic profiles. Individual subtypes, including cutaneous, acral, and mucosal, exhibit distinct mutational characteristics. Classic cutaneous melanoma is characterized by a high mutational burden associated with UV radiation exposure, making it highly immunogenic and rich in potential neoantigens. Conversely, the rarer acral and mucosal melanomas contain significantly fewer mutations, and their biology is dominated by chromosomal aberrations, limiting the number of possible immunological targets. This variability clearly demonstrates that a universal antigen panel will not be effective in all patients, reinforcing the importance of personalized mRNA vaccination.

Vaccines encoding tumor-specific neoantigens represent a highly promising therapeutic strategy because they direct the immune response against targets unique to a given tumor and are absent in healthy tissues. This approach minimizes the risk of autoimmunity while simultaneously enabling the immune system to “see” previously invisible mutations. However, the challenge lies in its practicality: developing a personalized vaccine requires sequencing the tumor genome, rapid bioinformatic identification of immunogenic mutations, and subsequent production of a personalized mRNA construct for each patient. This process remains costly and time-consuming, although ongoing technological improvements, including increasing automation, have significantly shortened production timelines, reducing them in pilot projects from approximately 9 weeks to less than four. Among the available technologies, the mRNA platform is considered the most time-efficient for developing personalized vaccines, offering hope for gradually overcoming financial and logistical barriers.

Despite numerous advantages, mRNA vaccines in oncology remain in the early stages of development. Key challenges include ensuring safety, optimizing the formulation’s immunogenicity and stability, and efficient delivery of mRNA to antigen-presenting cells. The risks of excessive activation of the innate immune system, increased cytokine production, and potential autoimmune reactions must also be considered. Furthermore, accurate selection of neoantigens based on point mutations or structural aberrations is crucial, requiring further development of sequencing technologies and bioinformatics tools.

Future directions for the development of mRNA vaccines in melanoma therapy include the development of more effective delivery systems that will increase the stability, protection, and penetration of mRNA into target cells, as well as the optimization of the combination of mRNA vaccines with other immunotherapeutic strategies, such as checkpoint inhibitors or adoptive T cell therapies. Further research on neoantigen panels will enable the development of more universal or semi-personalized constructs, shortening production time and reducing costs. Full realization of the potential of mRNA vaccines will require further technological improvements, integration with modern immunomodulatory strategies, and a deeper understanding of the biology of specific melanoma subtypes.

## Figures and Tables

**Table 1:** Registered studies on therapeutic mRNA vaccines in melanoma.

Trial Number	Treatment	Conditions	Status/Phase	Age (Years)	Locations
NCT02410733	Lipo-MERIT	Melanoma	Completed	≥18	Germany
NCT03897881	KEYNOTE-942	Melanoma	Recruiting	≥18	USA
NCT03313778	KEYNOTE-603	Melanoma	Active, not recruiting	≥18	USA, Australia, Japan, United Kingdom

## Data Availability

No new data were created or analyzed in this study.

## Abbreviations

The following abbreviations are used in this manuscript:

APC	Antigen-presenting cell
cDC1	Particularly cDC1
DOTAP	1,2-dioleoyl-3-trimethylammoniopropane
DOTMA	1,2-di-O-octadecenyl-3-trimethylammoniopropane
EVs	Extracellular vesicles
G antigen	GAGE
gp75	Glycoprotein 75
gp100	Glycoprotein 100
Melan-A/MART-1	Melanoma antigen recognized by T cells
LNP	Lipid nanoparticle
MAGE	Melanoma-associated antigen
mRNA	Messenger ribonucleic acid
MCSP	Melanoma chondroitin sulfate proteoglycan
MTf	Melanotransfetin
NRM	Nonreplicating mRNA
ORF	Open reading frame
PDT	Photodynamic therapy
ROS	Reactive oxygen species
SAM	Self-amplifying mRNA
TAA	Tumor-associated antigen
TAP	Transporter-associated with antigen processing
TME	Tumor microenvironment
TRP	Tyrosine-related proteins
